# Schizophrenia and Bipolar Polygenic Risk Scores in Relation to Intracranial Volume

**DOI:** 10.3390/genes13040695

**Published:** 2022-04-14

**Authors:** Sonja M. C. de Zwarte, Rachel M. Brouwer, René S. Kahn, Neeltje E. M. van Haren

**Affiliations:** 1 Department of Psychiatry, University Medical Center Utrecht Brain Center, Utrecht University, 3584 CG Utrecht, The Netherlands; r.m2.brouwer@vu.nl (R.M.B.); rene.kahn@mssm.edu (R.S.K.); 2 Department of Complex Trait Genetics, Center for Neurogenomics and Cognitive Research, Amsterdam Neuroscience, Vrije Universiteit Amsterdam, 1081 HV Amsterdam, The Netherlands; 3 Department of Psychiatry, Icahn School of Medicine at Mount Sinai, New York, NY 10029, USA; 4 VISN 2 Mental Illness Research, Education and Clinical Center, James J. Peters VA Medical Center, Bronx, NY 10468, USA; 5 Department of Child and Adolescent Psychiatry/Psychology, Erasmus University Medical Centre Sophia, 3000 CB Rotterdam, The Netherlands

**Keywords:** polygenic risk scores, bipolar disorder, schizophrenia, intracranial volume, UK Biobank, neurodevelopment

## Abstract

Schizophrenia and bipolar disorder are neurodevelopmental disorders with overlapping symptoms and a shared genetic background. Deviations in intracranial volume (ICV)—a marker for neurodevelopment—differ between schizophrenia and bipolar disorder. Here, we investigated whether genetic risk for schizophrenia and bipolar disorder is related to ICV in the general population by using the UK Biobank data (*n* = 20,196). Polygenic risk scores for schizophrenia (SZ-PRS) and bipolar disorder (BD-PRS) were computed for 12 genome wide association study *P*-value thresholds (*P*_T_) for each individual and correlations with ICV were investigated. Partial correlations were performed at each *P*_T_ to investigate whether disease specific genetic risk variants for schizophrenia and bipolar disorder show different relationships with ICV. ICV showed a negative correlation with SZ-PRS at *P*_T_ ≥ 0.005 (*r* < −0.02, *p* < 0.005). ICV was not associated with BD-PRS; however, a positive correlation between BD-PRS and ICV at *P*_T_ = 0.2 and *P*_T_ = 0.4 (*r* = +0.02, *p* < 0.005) appeared when the genetic overlap between schizophrenia and bipolar disorder was accounted for. Despite small effect sizes, a higher load of schizophrenia risk genes is associated with a smaller ICV in the general population, while risk genes specific for bipolar disorder are correlated with a larger ICV. These findings suggest that schizophrenia and bipolar disorder risk genes, when accounting for the genetic overlap between both disorders, have opposite effects on early brain development.

## 1. Introduction

Schizophrenia and bipolar disorder are two psychiatric diagnostic categories with overlapping symptoms, such as psychosis, anxiety, and depression [1]. In addition, they largely share a genetic background [2,3,4]. Structural brain imaging studies have shown that schizophrenia and bipolar disorder have different findings of intracranial volume (ICV); compared to controls, ICV is smaller in patients with schizophrenia [5,6,7] but not in patients with bipolar disorder [8]. Twin and family studies have reported that genetic liability for bipolar disorder, but not for schizophrenia, is associated with a larger ICV [9,10]. Thus, there is a double discrepancy between schizophrenia and bipolar disorder with regard to ICV, with smaller volumes in patients with schizophrenia as compared to controls but larger volumes in first-degree relatives of patients with bipolar disorder relative to controls.

Schizophrenia, bipolar disorder and ICV are all highly heritable [11,12,13], providing an opportunity to investigate whether the relationship between the disorders and ICV has a genetic origin, and if so, to what extent this is shared and/or distinctive for each disorder. While schizophrenia and bipolar disorder have a strongly overlapping genetic architecture [2,3,4], studies into the genetic overlap between these disorders and ICV have shown varying results. There is no evidence of a significant genetic correlation between either disorder and ICV based on the findings from genome wide association studies (GWAS) of schizophrenia and bipolar disorder [14,15]; however, genetic overlap between schizophrenia and ICV has been suggested through conditional false discovery rate analysis which identified shared loci [16], and through partitioning heritability analysis [17]. This suggests that alternative approaches to investigate the genetic relationship between schizophrenia, bipolar disorder, and ICV may provide additional information about the underlying shared and/or unique genetic relationship between the disorders and ICV.

One such approach is to use individual-level genotype data and estimate the correlation of polygenic risk scores of the disorders with ICV. A polygenic risk score of a disorder is calculated as a weighted sum of alleles that are associated with the disorder; it is a number that summarizes the estimated effect of many genetic variants on an individual’s phenotype. Thus, polygenic scoring allows us to investigate genetic relationships at an individual level, by linking a summed score of genetic risk loci for schizophrenia and bipolar disorder to an individual’s ICV. This approach also allows for investigating the effect of the polygenic risk scores on ICV in the general population, i.e., individuals who carry genetic loading for the disorders to some degree without being ill. The advantage of linking risk genes for psychiatric disorders to ICV in the general population is that, if a relationship is present, this may represent the effect of the risk genes on ICV without confounds of (premorbid) disease or potential gene-by-environment interactions. To date, no large studies in the general population have been conducted in which the effect of the polygenic risk scores for schizophrenia and/or bipolar disorder on ICV has been reported [18,19]. Due to the large genetic overlap between schizophrenia and bipolar disorder [2,3,4], a cross-disorder approach may provide crucial information to the shared and/or unique genetic contribution of polygenic risk for schizophrenia and bipolar disorder to ICV.

Here, we investigated to what degree the polygenic risk scores for schizophrenia (SZ-PRS) and bipolar disorder (BD-PRS) are related to ICV in the general population in 20,196 individuals from the UK Biobank. We hypothesized (i) a negative relationship between SZ-PRS and ICV; (ii) a positive relationship between BD-PRS with ICV, based on the double discrepancy in structural imaging findings, with smaller ICV in patients with schizophrenia and larger ICV in first-degree relatives of patients with bipolar disorder. To investigate whether disease-specific genetic contributions are related to ICV we computed partial correlations taking into account the genetic overlap between schizophrenia and bipolar disorder [2,3,4].

## 2. Materials and Methods

### 2.1. Study Sample

The UK Biobank dataset is a large prospective population-based cohort (https://www.ukbiobank.ac.uk, accessed on 15 February 2022). We included 20,196 participants (63.2 ± 7.4 years old age range 45.2 from 80.7); 52.5% female) with a good quality structural magnetic resonance imaging (MRI) scan and self-reported white ethnicity (i.e., white British, white Irish, and other white background). The UK Biobank has approval from the North West Multi-centre Research Ethics Committee (MREC) to obtain and disseminate data and samples from the participants (http://www.ukbiobank.ac.uk/ethics/, accessed on 15 February 2022), and these ethical regulations cover the work in this study. Written informed consent was obtained from all participants.

### 2.2. Image Acquisition and Processing

Structural T1-weighted MRI scans were acquired at three different scanner sites. FreeSurfer version 6.0 was used to obtain estimated Total Intracranial Volume (eTIV) in each individual (http://surfer.nmr.mgh.harvard.edu/, accessed on 15 February 2022). FreeSurfer calculates eTIV by dividing a predetermined constant with the factor by which the input magnetic resonance images are scaled in size to align to the MNI305 space (https://surfer.nmr.mgh.harvard.edu/fswiki/eTIV, accessed on 15 February 2022) [20].

### 2.3. Polygenic Scoring

Polygenic risk scores were calculated using schizophrenia- and bipolar disorder-associated alleles and effect sizes reported in the GWAS summary statistics [21,22]. Overlapping single-nucleotide polymorphisms (SNPs) between the GWAS (training dataset), 1000 reference Genome (reference dataset), and dataset of interest (target dataset) were selected. The following SNPs were excluded: (1) insertion or deletion, ambiguous SNPs; (2) SNPs with MAF < 0.01 and SNPs with imputation quality (R^2^) < 0.8; and (3) SNPs located in complex-LD regions [23]. The remaining SNPs were clumped in two rounds using PLINK (http://www.cog-genomics.org/plink/, accessed on 15 February 2022) [24]; round 1 with the default parameters (physical distance threshold 250 kb and LD threshold (R^2^) < 0.5; round 2 with a physical distance threshold of 5000 kb and LD threshold (R^2^) < 0.2; the resulting SNPs were used for polygenic score calculation. Odds ratios for autosomal SNPs reported in the summary statistics were log-converted to beta values. Polygenic scores were calculated using PLINK’s score function for 12 GWAS *p*-value thresholds (*P*_T_): 5 × 10^−8^, 5 × 10^−7^, 5 × 10^−6^, 5 × 10^−5^, 5 × 10^−4^, 0.005, 0.05, 0.1, 0.2, 0.3, 0.4, and 0.5.

### 2.4. Statistical Analyses

R version 3.5.0 (http://www.r-project.org, accessed on 15 February 2022) was used for statistical analyses. The first three principal components of genetic similarity, provided by the UK Biobank, were regressed out of each of the polygenic risk score. Age, sex, and scanner site were regressed out for the ICV measures. The residuals were used to calculate Pearson correlations between SZ-PRS, BD-PRS, for each *P*_T_, and ICV. Partial correlations were performed at each *P*_T_ to investigate whether disease specific genetic risk variants for schizophrenia and bipolar disorder risk show different relationships with ICV. The Matrix Spectral Decomposition (matSpD) approach was used to provide a measure of the equivalent number of independent variables (http://neurogenetics.qimrberghofer.edu.au/matSpD/, accessed on 15 February 2022) [25,26]. Ten independent variables were present in the correlation (*r*) matrix of SZ-PRS and BD-PRS combining all *P*_T_, therefore the significance threshold was set at *p* < 0.005 to control for multiple testing.

## 3. Results

As expected, BD-PRS and SZ-PRS were positively correlated for each *P*_T_, with correlations ranging from *r* = +0.10 at *P*_T_ = 5 × 10^−7^ to *r* = +0.41 at *P*_T_ = 0.5 (*p <* 0.005).

### 3.1. Relationship Schizophrenia Polygenic Risk Score and Intracranial Volume

**Figure 1:**
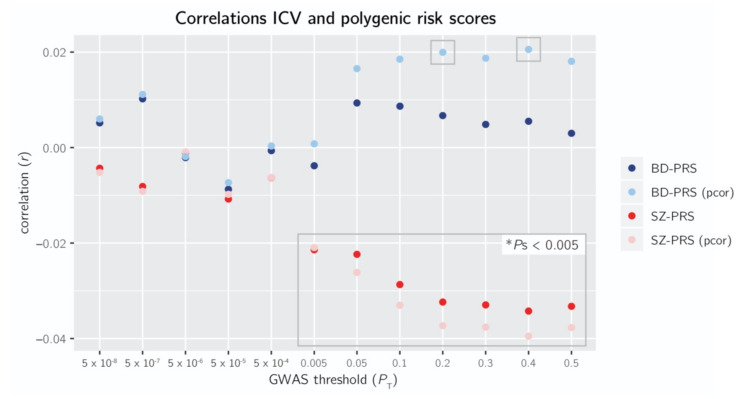
Pearson and partial correlations (pcor) of intracranial volume (ICV) with the polygenic scores of schizophrenia (SZ-PRS) and bipolar disorder (BD-PRS) at 12 genome wide association study (GWAS) *P*-value thresholds (*P*_T_): 5 × 10^−8^, 5 × 10^−7^, 5 × 10^−6^, 5 × 10^−5^, 5 × 10^−4^, 0.005, 0.05, 0.1, 0.2, 0.3, 0.4, 0.5. Gray boxes: *p* < 0.005.

SZ-PRS and ICV were negatively correlated at the more lenient GWAS thresholds *P*_T_ ≥ 0.005, ranging from *r* = −0.02 to −0.03 (*p* < 0.005; Figure 1). When accounting for BD-PRS, the negative correlation between SZ-PRS and ICV remained significant, ranging from *r* = −0.02 to −0.04 (Figure 1).

### 3.2. Relationship Bipolar Disorder Polygenic Risk Score and Intracranial Volume

No significant correlation was found between BD-PRS and ICV (Figure 1). However, when accounting for SZ-PRS, the positive correlation between BD-PRS and ICV reached significance at *P*_T_ = 0.2 and *P*_T_ = 0.4 (*r* = +0.02, *p* < 0.005; Figure 1).

## 4. Discussion

In this study we aimed to establish if, and to what degree, risk genes for schizophrenia or bipolar disorder are related to ICV by examining the relationship between ICV and the polygenic risk scores of each disease in the general population. ICV can be considered a marker of early neurodevelopment, since ICV grows exponentially in the first years of life, so that ~90% of its size is reached by the age of 5, while its growth is completed at age 15 [27,28]. This is well before the usual age of onset of most mental illnesses, such as schizophrenia and bipolar disorder [29,30,31]. Both illnesses have long been characterized as neurodevelopmental [32,33,34], although it has been proposed that abnormal neurodevelopment may play a larger role in the onset of schizophrenia than in bipolar disorder [35,36,37]. The large population sample of the UK Biobank allowed us to investigate the effects of these genes on ICV in the general population without potential effects of disease or disease-specific gene-by-environment interactions. We showed a negative association between SZ-PRS and ICV, while in contrast, the BD-PRS was associated with larger ICV when taking into account the disease-specific schizophrenia risk genes.

The finding of a small, yet significant, negative correlation between ICV and SZ-PRS, suggests that having a higher load of risk genes for schizophrenia leads to a slightly smaller ICV in the general population. The negative relationship between SZ-PRS and ICV is consistent with results from MRI studies showing that patients with schizophrenia [5,6,7], but also adolescents with early-onset psychosis [38] and individuals at clinical high risk for developing psychosis [39] have on average a smaller ICV than healthy individuals with effect sizes ranging between Cohen’s *d* −0.10 and −0.39. Given that ICV growth is completed around the age of 15, and that schizophrenia risk genes are related to smaller ICV in an adult population, this suggests that schizophrenia risk genes are involved in abnormal brain development early in life. Genetic overlap between schizophrenia and ICV based on conditional false discovery rate analysis and partitioning heritability analysis indeed has been suggested previously [16,17]. On the other hand, based on the absence of genetic correlations between schizophrenia and ICV [15] there is no direct evidence that the full range of common risk genes for schizophrenia are related to the genetic predisposition for the size of the ICV. This might imply that while genetic risk for schizophrenia leads to abnormal ICV development, schizophrenia risk genes are not the only genes involved in typical ICV development.

We found no significant correlation between BD-PRS and ICV in the general population. This finding corroborates results from clinical imaging studies, where no significant ICV differences between patients with bipolar disorder and controls have been reported [8]. Again, genetic correlations between bipolar disorder and ICV are small and non-significant [14]. Because, on a genome wide level, genetic variants explaining schizophrenia and bipolar disorder substantially overlap [40], we also investigated the partial correlations, taking the shared genetic risk between schizophrenia and bipolar disorder into account. This procedure allows us to investigate the contribution of disorder-specific genetic risk. When we accounted for SZ-PRS, a small significant positive relationship between BD-PRS and ICV emerged. We have previously shown that unaffected first-degree relatives of patients with bipolar disorder have larger ICV than both their ill family member and control individuals [10]. Combined with the positive relationship between bipolar-specific risk genes and ICV, that might suggest that the larger ICV in first-degree relatives may, in part, have a genetic background. In patients, a potential positive effect on ICV of genetic risk for bipolar disorder may be cancelled out by (premorbid) disease effects, making ICV a potential interesting target for predicting bipolar disorder in individuals in genetic high-risk populations (although predictive power is currently still very low).

The cross-disorder design of this study made it possible to directly compare between the relationship between ICV and risk for both schizophrenia and bipolar disorder, which potentially may lead to further insights into the shared and distinct neurodevelopmental background of both disorders. Our findings show that schizophrenia and bipolar disorder-specific risk genes have an opposite effect on ICV in the general population. That this divergent pattern became only apparent after accounting for the genetic overlap between the disorders highlights the importance of cross-disorder approaches. A recent study suggested that genetic risk loci for schizophrenia have been associated with psychotic features in bipolar disorder, explaining overlapping symptoms in these disorders [41]. In contrast, schizophrenia and bipolar disorder show neurodevelopmental differences in the cognitive domain [36], underlying the importance of studying both overlap and differences in genetic risk and symptomatology of schizophrenia and bipolar disorder.

It would be interesting as a follow-up study to investigate the relationship between ICV and genetic risk for illness in other disorders. There is evidence that ICV is linked to for instance autism, attention deficit hyperactivity disorder (ADHD), 22q11DS, clinical high risk for psychosis and early onset psychosis based on clinical meta- and/or mega-analysis studies in which brain imaging data of patients and controls were compared [38,39,42,43,44]. With the ever-increasing sample sizes of the latest GWASs, it would be interesting to see whether they show a relationship with ICV, to increase understanding of the role of very early development in the onset of different severe mental illnesses.

A few considerations to the current study should be noted. First, the correlations between genetic risk and ICV indicate a very small explained variance, that reaches significance only at relatively lenient *P*-value thresholds for inclusion of SNPs in polygenic scores. This confirms that we are still far from using polygenic scores as a tool to understand underlying biological measures at the level of the individual. In this cohort with individuals of the general population, the correlations reached only significance due the large sample sizes and therefore do not necessarily represent clinical relevance. Although our findings improve our knowledge about shared and distinct pathophysiological mechanisms related to schizophrenia and bipolar disorder, predictive power is currently insufficient for the use of early screening methods of for diagnoses. However, large correlations are not expected in the general population considering that the effect sizes for ICV deviations in patients with schizophrenia and first-degree family members of patients with bipolar disorder are modest at best [5,6,7,8]. In this study, we focused on ICV rather than to include other brain measures to estimate their relation to SZ-PRS and BD-PRS based on our previous findings. Moreover, gray and white matter abnormalities are often found to be related to illness or medication effects, while this is most likely less the case for ICV as it is fully developed at the age of 15, i.e., well before the usual age of onset. Nonetheless, a follow-up study investigating the relationship between the polygenic risk scores compared to other brain structures would be very interesting to further disentangle the effect of genetic risk on (early) brain development. Finally, in the UK Biobank population, the average age was 63 years old at the time of the MRI scan. A recent longitudinal study has shown that ICV is not constant during adulthood but instead shows small increases during young adulthood and decreases from the fourth decade of life [45]. It is possible that the relationship between ICV and SZ-PRS and BD-PRS is more pronounced earlier in life, before ageing effects take place. Important next steps may include investigating the development of ICV during childhood and adolescence in relation to genetic risk for schizophrenia and bipolar disorder and to repeat the current approach in large case-control or family design studies.

To summarize, we show a negative relationship between SZ-PRS and ICV in the general population, while BD-PRS and ICV are positively associated when taking into account the disease-specific schizophrenia risk genes. These findings suggest that schizophrenia and bipolar disorder-specific risk genes have an opposite effect on (early) brain development.

## Data Availability

The data presented in this study are openly available in the UK Biobank at: https://www.ukbiobank.ac.uk/, accessed on 15 February 2022. Summary statistics can be provided by the authors upon request.
